# Computerized Cognitive Behavioral Therapy to Treat Emotional Distress After Stroke: A Feasibility Randomized Controlled Trial

**DOI:** 10.2196/mental.6022

**Published:** 2017-05-31

**Authors:** Sara K Simblett, Matthew Yates, Adam P Wagner, Peter Watson, Fergus Gracey, Howard Ring, Andrew Bateman

**Affiliations:** ^1^ Institute of Psychiatry, Psychology and Neuroscience Department of Psychology King's College London London United Kingdom; ^2^ Department of Psychiatry University of Cambridge Cambridge United Kingdom; ^3^ National Institute for Health Research (NIHR) Collaboration for Leadership in Applied Health Research and Care (CLAHRC) East of England Cambridgeshire & Peterborough Foundation NHS Trust Cambridge United Kingdom; ^4^ Oliver Zangwill Centre for Neuropsychological Rehabilitation Ely United Kingdom; ^5^ Norwich Medical School University of East Anglia Norwich United Kingdom; ^6^ MRC Cognition and Brain Sciences Unit Cambridge United Kingdom

**Keywords:** cognitive therapy, technology, stroke, depression, anxiety

## Abstract

**Background:**

Depression and anxiety are common complications following stroke. Symptoms could be treatable with psychological therapy, but there is little research on its efficacy.

**Objectives:**

The aim of this study was to investigate (1) the acceptability and feasibility of computerized cognitive behavioral therapy (cCBT) to treat symptoms of depression and anxiety and (2) a trial design for comparing the efficacy of cCBT compared with an active comparator.

**Methods:**

Of the total 134 people screened for symptoms of depression and anxiety following stroke, 28 were cluster randomized in blocks with an allocation ratio 2:1 to cCBT (n=19) or an active comparator of computerized cognitive remediation therapy (cCRT, n=9). Qualitative and quantitative feedback was sought on the acceptability and feasibility of both interventions, alongside measuring levels of depression, anxiety, and activities of daily living before, immediately after, and 3 months post treatment.

**Results:**

Both cCBT and cCRT groups were rated as near equally useful (mean = 6.4 vs 6.5, *d*=0.05), while cCBT was somewhat less relevant (mean = 5.5 vs 6.5, *d*=0.45) but somewhat easier to use (mean = 7.0 vs 6.3, *d*=0.31). Participants tolerated randomization and dropout rates were comparable with similar trials, with only 3 participants discontinuing due to potential adverse effects; however, dropout was higher from the cCBT arm (7/19, 37% vs 1/9, 11% for cCRT). The trial design required small alterations and highlighted that future-related studies should control for participants receiving antidepressant medication, which significantly differed between groups (*P*=.05). Descriptive statistics of the proposed outcome measures and qualitative feedback about the cCBT intervention are reported.

**Conclusions:**

A pragmatic approach is required to deliver computerized interventions to accommodate individual needs. We report a preliminary investigation to inform the development of a full randomized controlled trial for testing the efficacy of computerized interventions for people with long-term neurological conditions such as stroke and conclude that this is a potentially promising way of improving accessibility of psychological support.

## Introduction

### Psychological Support Following Stroke

Emotional distress is a frequent complication following stroke. Approximately one-third of people report common mental health problems such as depression (33%) [1] or anxiety disorders (25%) [2] after experiencing a stroke. Longitudinal studies of poststroke depression and anxiety suggest that symptoms remain high throughout the acute and longer-term phases, for example, up to three years post stroke [3,4]. If left untreated, mental health problems have been found to significantly impact on functional recovery and quality of life [5].

Despite the clear need, it has been reported that relatively few people receive treatment for these commonly experienced mental health problems after stroke [1]. Stroke rehabilitation guidelines recommend routine clinical psychology input [6], but less than 40% of regions in the United Kingdom provide good access to psychological therapy [7]. This may in part be due to a relatively small evidence-base for treatment, especially psychological treatment, of depression and anxiety disorders following stroke [8,9]. Other barriers include the additional costs associated with providing psychological therapy and the difficulties service users may have in traveling to clinics for practical reasons such as physical and cognitive impairments, or lack of transport. This research sought to investigate the use of therapeutic technology, more specifically a computerized therapy package based on cognitive behavioral therapy (cCBT), as an accessible and, potentially, effective means of providing psychological treatment for common mental health problems following stroke.

### Computerized Therapy Interventions

Across populations, there is a weight of evidence toward computerized versions of CBT being effective treatments of depression and anxiety disorders. Indeed, recent meta-analyses conclude that cCBT could be a very promising and efficacious treatment for depression within a diverse range of settings and clinical groups [10] and for some specific anxiety disorders, that is, panic disorder and specific phobia [11] across urban and remote rural communities [12]. Furthermore, research has shown that cCBT has the potential to be as effective as therapist-delivered CBT [11,13]. Currently, the United Kingdom’s National Institute for Health and Clinical Excellence recommends cCBT as a possible treatment for depression among people with concurrent long-term physical health problems, such as neurological conditions including stroke [14]. However, there is a limited evidence-base for this recommendation.

In a review of the current research on the acceptability and feasibility of providing cCBT for people with a diagnosis of a neurological condition, including traumatic brain injury and multiple sclerosis, it was concluded that while cCBT has the potential to be of benefit, greater efforts are needed to improve the accessibility of such interventions to accommodate physical and cognitive difficulties [15]. This parallels research findings for the appropriateness of therapist-delivered CBT for clinical groups where physical and cognitive difficulties are more prevalent, for example, in older adult populations. CBT has been found to be effective at reducing symptoms of depression and anxiety among older adults but often requires adaptation and augmentation to accommodate individuals with more complex needs such as physical and cognitive difficulties [16,17]. Evidence drawn from these studies, along with a systematic review of the evidence for use of cCBT with older adults [18], suggests that it may be necessary to provide users with a greater level of support to complete the cCBT programs. This is supported in the general literature on cCBT, which has concluded that “guided” cCBT yields better outcomes [10]. Further barriers for accessing cCBT and psychological support, in general, include the perceived social stigma attached to seeking support from mental health services [19] and associated additional immediate costs, despite potential long-term payoffs [20].

To summarize, the work here contributes to the goal of making psychological support for symptoms of depression and anxiety more accessible for people who have experienced a stroke. In particular, we explored the acceptability and feasibility of a particular intervention for symptoms of depression and anxiety, in preparation for testing its efficacy in a larger randomized controlled trial (RCT). We investigated the feasibility of providing access to computerized therapy interventions embedded within people’s local communities. Furthermore, we report on a pilot RCT of a cCBT intervention (referred to throughout as the “active condition”) as compared with an alternative (“control”) condition. Both involved active engagement in structured activities via a computer. The alternative “control” activity was loosely based on a computerized cognitive remediation therapy (cCRT) approach and focused on practicing cognitive skills in a series of training exercises as opposed to addressing mood directly. An active comparator condition was designed to directly assess efficacy related to the content of the treatment intervention. This work followed guidelines for conducting and reporting on the feasibility of randomized pilot studies [21], with the view of progressing toward the design of a larger scale RCT to assess the effectiveness of the therapeutic technology, for example, cCBT, as an addition to current practice in community stroke and other neurorehabilitation services.

## Methods

### Participants

We recruited 134 people who had been medically assessed and diagnosed as having experienced a stroke within the last 5 years (from April 2011 to April 2012) and had not subsequently received a diagnosis of a neurodegenerative condition (eg, dementia) from three community-based neurorehabilitation services situated in a large county, consisting of a mix of rural and urban areas. All were screened for symptoms of depression and anxiety using the Beck Depression Inventory-II (BDI-II) [22] and Beck Anxiety Inventory (BAI) [23], and 28 people scoring high on these measures (as detailed in Textbox 1) consented to take part in a pilot RCT. The full inclusion and exclusion criteria are shown in Textbox 1. Ethical approval was granted by the local National Health Service (NHS) Research Ethics Committee (reference no.: 10/H0311/62).

### Interventions

Participants completed an eight-module course of either cCBT or an active comparison condition in addition to their usual care, which included general practitioner (GP) support and in some cases antidepressant medication.

cCBT condition: the first group completed a computerized package (also available in an Web-based format over the Internet) called “Beating the Blues” (formerly Ultrasis Plc), which was developed to treat symptoms of depression and anxiety following principals drawn from CBT. At the time of the study, this package had the best evidence base for treatment of symptoms of depression within primary care settings compared with other computerized packages available [24], and following the success of a pilot clinical case study [25] the idea was to see whether it could also be a feasible and acceptable “off the shelf” intervention for people who had experienced a stroke.

Active comparison condition: the second group completed an intervention designed to be an active comparator for the cCBT condition using Web-based resources. This involved a series of training exercises aimed at rehearsing cognitive skills, including memory, attention, visuospatial, and executive functioning, considering compensatory strategies where possible. It was loosely based on CRT, defined by consensus as “a behavioural-training based intervention that aims to improve cognitive processes (attention, memory, executive function, social cognition, or metacognition) with the goal of durability and generalization” [26].

Specific details about the structure and content of the two interventions are provided in Multimedia Appendix 1.

**Participant inclusion and exclusion criteria.**

Inclusion criteria

- Aged 18 years and over
- Experienced a stroke within the last year
- Mild or moderate depression or anxiety defined by the Beck Depression Inventory-II (BDI-II) [22] score >13 or the Beck Anxiety Inventory (BAI) [23] score >7 or endorsement of being often bothered by feeling down, depressed, or hopeless, and having little interest or pleasure in doing things in the last month

Exclusion criteria

- Unable to give full informed consent to participate in the research
- A diagnosis of a neurodegenerative condition (eg, vascular dementia) or a symptomatic acquired brain injury, other than stroke
- A visual or auditory problem that could not be corrected and would seriously interfere with the participation in the research study
- Unable to undergo a verbal interview due to impairment of comprehension (including severe receptive aphasia)
- Currently severely depressed or reporting active suicidal ideation defined by BDI-II score ≥29 or BDI-II item 9 (suicidal ideation) score ≥2

Currently receiving psychological therapy or antidepressant medication for treatment of a mood or anxiety disorder

A number of common factors were shared between the way in which the two interventions were delivered. Where possible, participants completed one module per week, which lasted approximately one hour for a series of eight consecutive weeks. Each participant made use of a computer to administer the treatment and received guidance from a researcher with a master’s level degree in neuropsychology and supervision from a clinical neuropsychologist to facilitate engagement in the scheduled computerized activity. Both interventions took place in community-based, nonclinical settings. Participants in both conditions were first encouraged to complete the interventions on computers provided in local libraries or laptops (one of four) provided by the researchers in other community-based settings, for example, village halls. Participants were invited to attend these sessions in small groups at a mutually convenient time. A professional was present at all times and participants worked independently on their own computer. The idea of small groups was primarily introduced as a means of improving the feasibility of delivery, for example, reducing the costs associated with providing psychological therapy. However, if this was not possible, for example, where participants could not attend a community-based location, the researchers provided individualized support either remotely via telephone or email, or face-to-face within the participant’s home, bringing a laptop where needed. All participants received a combination of telephone and email support in between computerized treatment sessions to further facilitate engagement in the treatment interventions.

### Treatment Allocation

Participants were randomized in clusters into cCBT and active comparator conditions using a ratio of 2:1, that is, for every one cluster randomized into the control condition, two clusters were randomized into the cCBT condition. The use of this ratio allowed for analysis of the feasibility of a larger scale RCT in this area with a greater focus on assessing the appropriateness, acceptability, and relevance of the cCBT intervention. Randomization blocks of three (1 × cCRT, 2 × cCBT), six (2 × cCRT, 4 × cCBT), and nine (3 × cCRT, 6 × cCBT) were applied in a randomly generated sequence to help balance the number of participants receiving each of the two conditions. This allocation sequence was generated by a researcher external to the research team and saved in a location where it could not be accessed by any of the researchers involved in the study. In order to allocate a participant to a condition, a member of the research team had to send an email to the person with access to the allocation sequence, specifying a cluster number. The prespecified condition that corresponded to the cluster number was then returned via email.

Clustering was determined pragmatically, on the basis of the region or area in which the participant lived and the ease of attending a group at one of several localities. As soon as two people were available within the same region and they both provided consent to take part in the study, they were randomized into one of the two conditions. If more than two people within the same region became available at the same time, pairings were determined by assigning each person a number and allocating them to a cluster according to a randomly generated list of numbers (ie, the numbers that appeared first and second in the sequence were grouped together, the two numbers that appeared third and fourth in the sequence were grouped together).

### Data Collection

In line with this being a pilot study, we focused on the feasibility and acceptability of the active intervention and the proposed study design. Acceptability was measured using ratings of appropriateness, usefulness, and ease of use for each computerized module on an 8-point scale with higher scores indicating a greater level of satisfaction with the intervention. Information on variables such as number of weeks spent in the intervention phase; number of treatment modules (out of a total of eight) completed; and the proportion of sessions completed in a group, in a community-based setting outside of their own home and with the additional face-to-face support of a clinical helper were collected to inform the feasibility of the current intervention design for this sample. Reasons for dropout were also gathered, where possible. Recruitment ended a year following commencement of data collection.

Participants also completed several quantitative outcome measures, including the BDI-II and BAI, as well as a measure of participation in activities of daily living (the Nottingham extended activities of daily living scale, NEADLS) [27]. The outcome measures were collected at baseline, just after completion of the last module (or when a participant decided to stop treatment early), and 3 months later.

### Data Analysis

#### Feasibility and Acceptability

Analysis of feedback on the feasibility and acceptability of the interventions was descriptive, reporting on the means or median values, and standard deviations or interquartile ranges, and also involved *t* tests to assess group differences. This information was used to address the following questions.

For the feasibility of the research and intervention protocols, the questions were as follows:

- Recruitment: how many people were screened and enrolled each month?
- Adherence: were both treatment interventions able to be delivered as specified in the intervention and research protocol? This included following the intended length, setting, and format of the interventions, as well as following the randomization and assessment procedures specified.
- Differences between trial arms: were there any deviations from the research protocol that were specific to either of the intervention conditions? Were these differences statistically significant?

For the acceptability and relevance of the interventions, the questions were as follows:

- Subjective ratings: Did the majority of participants report that the interventions were relevant to their problems, easy to engage in, and useful?
- Reasons for dropout: How many people dropped out and did not complete the entire intervention? Did participants who dropped out report any adverse effects?

#### Self-Reported Symptom and Activity Outcomes

Descriptive statistics of the outcome measures (BDI, BAI, and NEADLS) from the three measurement points are also reported.

## Results

### Feasibility of the Research and Intervention Protocols

#### Recruitment

In the early stages of recruitment, it became clear that fewer people than expected met the rigorous inclusion criteria set out in Textbox 1. For example, the recruitment rate for the intervention ran on average at 0.31 of the expected rate (n=8 per month) during the first two months. In part, this was due to fewer than expected people being available for initial screening (recruitment rate = 0.32). Thus, two alterations were made: first, the recruitment procedure for screening was widened to include people who had experienced a stroke during the last *five* years (as opposed to within a year of stroke). Second, the eligibility criteria for the intervention phase were further relaxed to include people who were currently taking a stable dose of antidepressant medication (as opposed to only those using *no* antidepressant medication); stable was defined as no modification within the 8 weeks prior commencing participation. These changes highlighted an unmet need: while we set out to treat symptoms of depression, we ended up treating co-morbid problems with depression and anxiety up to five years post stroke, a proportion of whom were receiving medical intervention but no psychological input to treat mood before commencing participation in this study.

**Figure 1:**
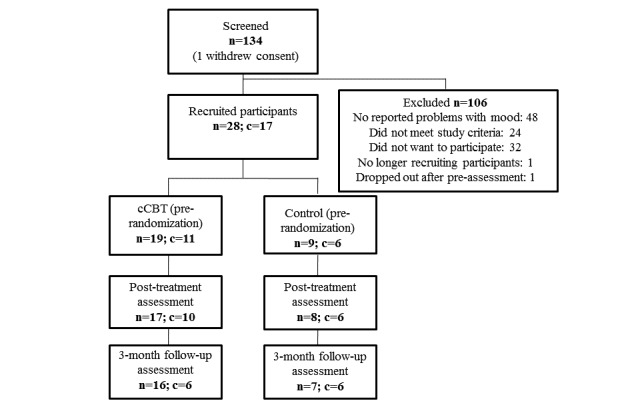
Flowchart of participants included in each phase of the study. n=number of individual participants, c=number of clusters, cCBT=computerized cognitive behavioural therapy, cCRT=computerized cognitive remediation therapy.

#### Adherence

Of the 134 screened potential participants, 28 met the inclusion criteria and were randomly allocated into the cCBT (n=19) or cCRT (n=9) intervention. Figure 1 details the number of participants who were recruited and assessed at baseline, immediately post treatment and at the 3-month follow-up time points for the two groups. Both treatment interventions were able to be delivered as intended, via a computer. However, while group comparisons were underpowered to detect statistically significant differences, effect size estimates suggested small differences in the delivery of the intervention with participants belonging to the cCBT group spending, on average, a greater number of weeks in the intervention and attending a larger number of sessions (see Table 1). However, participants allocated to the cCRT group completed the intervention within a time that was closer to the intended treatment protocol (8 weeks). This may be accounted for by the greater number of face-to-face sessions scheduled at regular weekly intervals by the researcher for participants in the cCRT group (9/9, 100%) as compared with the cCBT group (16/19, 84%), which was accessible online and able to be completed independently.

In general, the complexity of both of the interventions and the heterogeneous needs of the sample in relation to cognitive, functional, and physical functioning, as well as familiarity with use of computers, meant that there was a fairly high degree of variation in how the research protocol was applied between participants. Of note was the difference in time from discontinuation of treatment and administration of the post-assessment between the two groups (cCBT: mean 31 days [SD 31 days]; cCRT: mean 8 days [SD 6 days]).

Some individuals needed more support (eg, face-to-face technical assistance) to complete the computerized interventions. For example, a proportion of participants found it difficult to remember to do the homework tasks; some were able to do tasks between sessions if a carer prompted them to complete the exercises; others reported that they could remember to do the homework tasks but felt unmotivated or unable to do so.

Furthermore, although it had been initially intended that participants would complete the computerized intervention in groups, the median group size was only two people, and this was only achieved for 40% of cCBT sessions completed and 32% of cCRT sessions completed. Several participants (cCBT: 6/19, 32%; cCRT: 5/9, 56%) completed the intervention in their own homes on their own computer or a computer that was provided by the research team.

**Table 1:** Raw (unadjusted) differences between baseline characteristics of participants included in the two treatment arms, separately.

Variable	cCBT^a^	Active control, cCRT^b^	Group differences
N	19	9	
Female, n (%)	9 (47)	1 (11)	Fisher exact test, *P*=.10 *OR*=7.20
Age, mean (SD^c^)	62.1 (11.4)	64.6 (8.1)	Dif^d^=2.5 (95% CI −5.3 to 10.3), *P*=.51, *D*^e^=0.27
Time since stroke, median (IQR^f^: 25th-75th)	1.190 (0.5-1.1)	0.89 (0.6-4.1)	Kruskal-Wallis χ^2^_1_ = 2.2, *P*=.14
GCSEs^g^, n (%)	7(37)	7 (78)	Fisher exact test, *P*=.16, *OR*^h^=4.50
A-levels, n (%)	9 (47)	2 (22)	
Bachelor’s, n (%)	3 (16)	0 (0)	
Master’s, n (%)	0 (0)	0 (0)	
PhD, n (%)	0 (0)	0 (0)	
Taking anti-depressants, n (%)	10 (53)	1 (11)	Fisher exact test, *P*=.05, *OR*=8.89
Baseline BDI-II^i^, mean (SD)	19.1 (5.8)	13.4 (4.1)	Dif=−5.6 (95% CI−9.6 to −1.6), *P*=.01, *D*=1.19
Baseline BAI^j^, mean (SD)	11.2 (7.6)	8.3 (6.2)	Dif=−2.8 (95% CI−8.5 to 2.8), *P*=.31, *D*=0.42
Baseline NEADL^k^, mean (SD)	45.5 (14.6)	53.6 (12.5)	Dif=8.0 (95% CI−3.2 to 19.3), *P*=.15, *D*=0.61
Sessions attended, mean (SD)	6.3 (2.5)	7.2 (2.3)	Dif=1.0 (95% CI −1.1 to 3.0), *P*=.34, *D*=0.40
Weeks in intervention phase, mean (SD)	11.3 (7.7)	8.9 (3.3)	Dif=−2.4 (95% CI −6.7 to 1.8), *P*=.25, *D*=0.47

^a^cCBT: computerized cognitive behavioral therapy.

^b^cCRT: computerized cognitive remediation therapy.

^c^SD: standard deviation.

^d^Dif: difference.

^e^*D*: Cohen effect size measure.

^f^IQR: interquartile range (25-75).

^g^GCSE: General Certificate of Secondary Education.

^h^Odds ratio based on differences between controls and CBTs on just GCSEs and A-levels.

^i^BDI-II: Beck Depression Inventory-II [22].

^j^BAI: Beck Anxiety Inventory [23].

^k^NEADL: Nottingham extended activities of daily living [27].

#### Differences Between Trial Arms

There were also deviations to the research protocol in terms of the characteristics of participants allocated to the two intervention conditions. By design, as described in the methods, the cCBT arm had twice as many participants as in the active control arm. Significantly more (*P*=.05) of the cCBT group (10/19, 53%) were taking antidepression medication than in the control group (1/9, 11%), a large effect (odds ratio, OR=8.89). This may be linked with the significantly (*P*=.008) higher average level of baseline BDI-II score in the cCBT group (mean 19.1) than in the control group (mean 13.4). Raw unadjusted differences between the trial arms are shown in Table 1. Additional information on the characteristics of the participants included in the two trial arms in terms of their functional, cognitive, and estimated premorbid intelligence quotient abilities are provided in Table 2. Group comparisons were underpowered due to small sample sizes, and although none of the differences in Baseline BAI, Baseline NEADL, Sessions attended, and weeks in intervention phase between the two arms reached statistically significant differences, comparisons did still attain a moderate effect size as shown in Table 1.

### Acceptability and Relevance of the Interventions

#### Subjective Ratings

**Figure 2:**
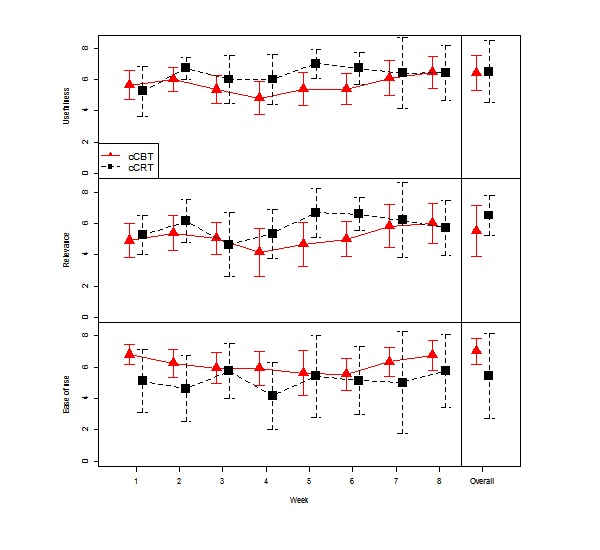
Ratings of usefulness, relevancy, and the ease of use of each session and the courses overall for (1) computerized cognitive behavioral therapy (cCBT) and (2) computerized cognitive remediation therapy (cCRT) as a comparison condition.

Average ratings of the usefulness, relevancy, and the ease of use of each session of the two interventions are shown in Figure 2. These ratings ranged from 4 to 7 out of 8 for useful, relevant, and easy to use across participants for all sessions. Both cCBT and cCRT were rated as near equally useful (mean=6.4 vs 6.5, *d*=0.05), while cCBT was somewhat less relevant (mean=5.5 vs 6.5, *d*=0.45) but somewhat easier to use (mean=7.0 vs 6.3, *d*=0.31). Group comparisons were underpowered due to small sample sizes and none of these differences in the overall ratings between the two arms reached statistical significance (usefulness: *P*=.93; relevancy: *P*=.38; and the ease of use: *P*=.13).

**Table 2:** Functional ability, cognitive functioning, and estimated premorbid intelligence quotient (IQ) of participants included in the two intervention arms.

Functional Domain		cCBT^a^ (n=19)	Active control, cCRT^b^ (n=9)
**Functional ability, raw scores on NEADLS: mean (SD^c^), min=0**			
	Mobility, max=9	6.26 (2.51)	7.11 (2.15)
	Kitchen activities, max=7	5.68 (1.77)	6.78 (0.44)
	Other domestic activities, max=6	3.42 (1.87)	4.46 (2.40)
	Leisure activities, max=9	5.59 (2.17)	6.22 (2.49)
**General cognitive functioning, raw scores on ACE-R^d^: mean (SD), min=0**			
	Attention/orientation, max=18	17.74 (0.56)	17.11 (2.03)
	Memory, max=26	19.11 (6.00)	21.56 (3.71)
	Verbal fluency, max=14	9.05 (3.44)	10.00 (3.87)
	Visuospatial skills, max=16	14.21 (2.02)	14.78 (1.09)
	Language, max=26	23.74 (1.33)	22.22 (4.29)
	Overall cognition, max=100	84.21 (9.70)	84.21 (9.11)
Estimated premorbid IQ, from NART^e^ raw scores: mean (SD), min=0		112.11 (4.46)	109.78 (7.58)

^a^cCBT: computerized cognitive behavioral therapy.

^b^cCRT: computerized cognitive remediation therapy.

^c^SD: standard deviation.

^d^ACE-R: Addenbrooke’s cognitive examination [28].

^e^NART: National Adult Reading Test [29].

#### Reasons for Dropout

Across the course of the intervention, more participants dropped out of the cCBT (7/19, 37%) as compared with the comparison (1/9, 11%) condition as shown in Table 3. One participant dropped out before starting treatment due to improved mood. Due to other commitments, a participant from the cCRT group dropped out before posttreatment assessment. Posttreatment assessment data for two participants in the cCBT group were lost to follow-up (see Figure 1). Of most concern were those who dropped out due to potential adverse effects of the intervention, all of whom belonged to the cCBT condition. These individuals reported slightly worse mood or more anxiety as a consequence of commencing cCBT. No one reported additional risks (eg, thoughts to harm self) as a result of completing modules in the cCBT condition. Any potential adverse outcomes were notified to participants’ GPs and, with permission, they were referred on to a clinical psychologist specializing in neurorehabilitation.

### Descriptive Statistics for Self-Reported Symptoms and Activity Outcomes

Table 4 displays the descriptive scores across the three different time points for both groups. All groups demonstrated a decrease in symptoms of distress across time, but there was very little difference in terms of functional ability between pre- and postintervention measurement points. A larger sample of participants is needed to establish reliable magnitudes of change or to measure group differences.

**Table 3:** Number of people who dropped out from one or other of the treatment conditions and their reasons for this.

Reason for dropout	cCBT (n=19)	cCRT (n=9)
Other commitment, n (%)	2 (10.5)	1 (11.1)
Potential adverse effect, n (%)	3 (15.8)	0 (0)
Ineffective, n (%)	1 (5.3)	0 (0)
Deterioration not due to intervention, n (%)	1 (5.3)	0 (0)

**Table 4:** Descriptive statistics for the three repeated measures at the three time points.

Time		Beck Depression Inventory (BDI-II)						Beck Anxiety Inventory (BAI)						Nottingham extended activities of daily living (NEADL)					
		cCBT^a^			cCRT^b^			cCBT			cCRT			cCBT			cCRT		
		N	Mean (SD^c^)	95% CIs	N	Mean (SD)	95% CIs	N	Mean (SD)	95% CIs	N	Mean (SD)	95% CIs	N	Mean (SD)	95% CIs	N	Mean (SD)	95% CIs
Baseline		19	19.1 (5.8)	16.3-21.9	9	13.4 (4.1)	10.3-16.6	19	11.2 (7.6)	7.5-14.8	9	8.3 (6.2)	3.6-13.1	19	45.5 (14.6)	38.5-52.6	9	53.6 (12.5)	43.9-63.2
Postintervention		15	9.5 (8.1)	5.0-14.0	8	9.4 (5.6)	4.7-14.1	15	8.7 (9.0)	3.7-13.7	8	6.5 (6.1)	1.4-11.6	14	52.4 (12.0)	45.5-59.4	7	54.6 (13.5)	42.1-67.1
Three-month follow-up		15	7.9 (5.3)	4.9-10.8	7	11.1 (6.8)	4.8-17.4	16	9.3 (11.4)	3.2-15.4	7	6.1 (4.3)	2.2-10.1	15	49.1 (15.8)	40.3-57.8	6	53.5 (18.1)	34.5-72.5

^a^cCBT: computerized cognitive behavioral therapy.

^b^cCRT: computerized cognitive remediation therapy.

^d^SD: standard deviation.

## Discussion

This study reports on the feasibility and acceptability of a cCBT intervention compared with an alternative computerized therapy condition based on CRT. Overall, our protocol design was reasonable: both interventions were considered appropriate, accessible, and useful. However, a number of adaptations were required to the research protocol and it was clear that a pragmatic approach is required to deliver computerized interventions to accommodate individual needs, the specifics of which will be discussed.

### Feasibility of the Research and Intervention Protocols

In terms of the feasibility of the research and treatment protocols, most aspects were followed, as planned. Indeed, all people who enrolled into the intervention phase were able to access the resources needed to engage in a computerized psychological intervention. This was achieved by a flexible and pragmatic approach to service provision, with participants using a combination of home-based and other community-based (eg, library) computers. However, some aspects of the intervention protocols required a greater level of flexibility; this included an extension to the length of time needed for participants to complete the intervention and allowing a proportion of participants to access the interventions independently. This was, in part, due to the geographically dispersed area over which the interventions were carried out and the heterogeneous needs of the population in terms of cognitive, communication, and physical abilities. Some of the deviations to the research protocol were in line with previous findings [30], in which a sample of people who had experienced a traumatic brain injury also took longer than expected to complete a course of cCBT. These authors suggest that this was a reflection of limitations posed by cognitive difficulties and that many people found it hard to access necessary computer and Internet resources. The cCBT intervention, which enabled participants to log on remotely, over the Internet, without the assistance of a researcher to facilitate this, made for greater accessibility. However, overall, participants in the cCBT group took longer to complete the intervention when they accessed the program independently with remote supervision. Although this meant that they also completed a greater number of sessions, there are implications for resources such as the length of time that online interventions are and remote supervision is made available to clients.

In terms of practical considerations, without access to a printer, participants needed to be provided with hard copies of materials, some of which were required in order to record completed tasks in daily-life between sessions. The need for “off-line” resources may have contributed to difficulties with adhering to this part of the intervention protocol, for example, completion of tasks between sessions. It suggests a benefit for interventions that can be completed entirely computerized or administered “online” via either a computer or mobile device. This suggestion will be important to consider when developing further resources. Although it is important to note that preferences for a “high-tech” treatment may naturally be more acceptable to some as compared with others, and perhaps relates to previous familiarity with technology, this hypothesis was not formally assessed in this study.

The computerized control intervention worked well as a comparator intervention, but required a greater degree of technical facilitation and shared a number of “active components” with the cCBT intervention, such as therapeutic contact and activity scheduling, both of which could have a potential impact on mood, for example, behavioral activation has been shown to be an effective intervention for reducing symptoms of depression [31]. Future studies could consider including a “treatment as usual” condition, such as a waiting-list design. The differences in characteristics between the two trial arms is also important for the purpose of designing future experimental studies and clinical trials to test the efficacy of computerized or Web-based psychological resources, which was beyond the scope of this study. In a larger scale RCT, certain variables (eg, severity of symptoms at baseline) should be more balanced with a greater sample size. However, consideration of stratifying, for example, by antidepressant use, will be worth noting for the design of future RCTs.

It was intended that postintervention assessment would be carried out by someone who was blind to which intervention participants had received. Unfortunately, this did not prove to be feasible due to limited resources. Although, where possible, the postintervention assessments were sent to participants in the post (self-report measures only) and were completed without supervision from a researcher, in an attempt to reduce observer bias, this methodological issue should be considered in greater depth when designing a larger scale trial.

A final, interesting, and somewhat unexpected finding with regard to feasibility was the discovery of a significant minority of people who had ongoing problems with anxiety and depression beyond the first year following their stroke. These people were almost invariably not receiving any support from neurorehabilitation services and their management was being primarily overseen by their GP. This demonstrates a potential unmet need in current service provision for emotional support following a stroke, and it makes a case for longer availability of psychological input *and better collaboration between psychological services and primary care*.

### Acceptability and Relevance of the Interventions

Quantitative ratings of usefulness, relevance, and ease of use of the treatment sessions and intervention conditions overall were a useful addition to this study over previous feasibility studies in this area [30,32]. They demonstrated that the majority of the content was deemed useful for the population. However, the cCBT was rated as somewhat less relevant but somewhat easier to use. There were also variations both across sessions and across participants that could be used to guide the development of future resources that could be targeted more to the needs of this specific clinical group.

It is encouraging to note that many of the participants in the cCBT condition provided very positive qualitative feedback about the package. In support of the quantitative ratings, people recognized the usefulness of the content; a participant fed-back that they “could see how it relates directly to time management, i.e. identify tasks and priorities and set aside (a) date and time to complete them,” which they self-identified as “something I have an issue with since I am at home 24/7 and as such feel I can do anything any time and struggle to be disciplined.” A number of people commented that cCBT helped to improve their level of confidence, feel more positive about the future, and less frustrated. One participant commented:

I do feel as if I have strategies for coping now. I just have to keep reminding myself to use them. The program did help me a lot and I believe it has helped me cope with a lot of the inner beliefs about myself that were not quite accurate.

Another participant, who particularly benefited from cCBT, expressed that:

It’s helped me come out of my comfort zone and face things, and that it helps you to understand yourself because you feel different.

Previously, this participant had described experiencing a stroke as “it’s like an alien creeping in one side of you.” The same participant told the facilitator that they had used the worksheets to help communicate how they were feeling with their family and friends. This was an unexpected positive finding.

The dropout rate in the present study for the cCBT intervention was almost identical to that reported in a previous study [30] (37% as compared to 38%, respectively) and is also comparable to findings reported in other studies of cCBT within the general population [24]. While some reasons for dropout were primarily practical, for example, other time commitments, again, in support of the results of the qualitative ratings, a degree of dissatisfaction related to the relevance of the content of the cCBT program was raised. One participant explained that:

My anxiety, when it happens, is caused by frustration of not being able to do simple things easily and then getting angry over it; for me, depression is too strong a description for how I feel, but unhappy, angry and annoyed, definitely; I find the exercises difficult since I do not go through anxiety or depression which the course is aimed at.

Another participant mentioned that they thought that their difficulties related to low self-esteem, rather than depression or anxiety. It is worth highlighting that the dropout rate for the cCRT group was lower than for the cCBT group (11% as compared with 37%). The reason for this is unknown and may reflect differences in the method of delivery between the two groups with a larger proportion of participants in the cCRT group receiving face-to-face support in their own homes. However, it is also possible that this result indicates that the cCRT intervention was more acceptable to the participants who undertook it.

Despite some people responding well to the interventions trialed in this study, others reported feeling worse (ie, reported greater levels of anxiety and lower mood) as a consequence of starting to complete the computerized therapy courses, specifically in relation to the course of cCBT. In general, little was known about other life circumstances that may have contributed to an increase in psychological distress. Therefore, more information is required before concluding whether or not participants reporting an increase in scores on the outcome measures experienced an adverse effect specific to the intervention received. Further follow-up, including more in-depth interviews, could be useful in answering this question. However, potential risks associated with any intervention must also be addressed. This highlights the need to consider the relevance of the clinical intervention recommended, given each person’s individual situation and presenting problems, and for suitable procedures to be in place to allow for escalation beyond low intensity interventions such as cCBT to access a greater level of psychological support, where necessary. Variation in response could be accounted for by differences in cognitive abilities. Indeed, research has found that executive functioning moderates response to CBT for generalized anxiety within an older adult population [33]. Research suggests augmenting CBT interventions with techniques to promote internal motivation to make behavioral changes, directly addressing issues associated with grief and loss as well as accommodating to cognitive abilities so that they are individually tailored to a person’s needs following a stroke [34].

### Future Research and Conclusions

Further work is needed to target computerized or other Web-based self-help interventions such as cCBT to the right people. There is good evidence to support the effectiveness of cCBT for depression [10,35]. However, depression might not have been the primary problem for everyone. Indeed, it has already been mentioned that a mixed group of people displaying problems commonly associated with depression and/or anxiety were recruited to take part in this study, some of whom may have required greater, or different, support for anxiety. This study trialled the feasibility and acceptability of an “off the shelf” cCBT package to treat symptoms of depression and anxiety; there is scope to develop a Web-based intervention that is more targeted to the specific therapeutic needs of people post stroke, which also takes into account the practical limitations they may face, such as physical restrictions, and the need to rely on carers for transport. Similarly, people’s ability to access and use the technology necessary to run the software should be considered. In this study, several people were guided to learn how to use a computer as part of the intervention, demonstrating that this is possible to achieve but requires adequate technical support.

From a broader perspective, it is important to consider barriers that impact the use of cCBT within health services. When exploring the infrastructure and information technology (IT) policies of the NHS in the United Kingdom, it was found that service users are limited by the number of computers they have access to [36]. It has also been highlighted that IT policies restricted the ability for NHS staff to provide ongoing guidance and support to potential cCBT users through, for example, contact via service user’s personal email account. Moreover, for cCBT to be an effective alternative to face-to-face therapy, the perceptions of service providers must be considered. Although previous research has reported good general acceptance of Web-based psychotherapy [37], other evidence suggests that health care professionals hold negative perceptions of cCBT, and this could impact its uptake [38]. For cCBT to be a viable and feasible intervention it is important that health services implement robust and streamlined IT infrastructures and provide information, training, and support to their staff so as not to cause additional clinical burden or interrupt therapeutic relationships [39]. These factors will be important for enabling generalization of findings reported in this study.

Despite these challenges, computerized therapy packages such as cCBT offer a promising means of making psychological support more accessible to people who have experienced a stroke. Further research, in an appropriately powered RCT, is needed to determine the efficacy of the cCBT interventions over and above other treatment options and the process of natural recovery. However, this study has demonstrated that guided cCBT is a feasible and appropriate intervention for many people who have experienced a stroke and, if found to be effective for treating symptoms of depression and/or anxiety, it could be a useful tool to add to the repertoire of neurorehabilitation services, increasing access to psychological support.

## Appendix

Multimedia Appendix 1 Summary of the structure and content included in the two interventions.

## Abbreviations

ACE-R: Addenbrooke’s cognitive examination
BAI: Beck Anxiety Inventory
BDI-II: Beck Depression Inventory-II
cCBT: computerized cognitive behavioral therapy
cCRT: computerized cognitive remediation therapy
CLAHRC: Collaboration for Leadership in Applied Health Research and Care
EoE: East of England
GCSE: General Certificate of Secondary Education.
GP: general practitioner
IT: information technology
NART: National Adult Reading Test
NEADLS: Nottingham extended activities of daily living scale
NHS: National Health Service
NIHR: National Institute for Health Research
SD: standard deviation

## References

[1] Hackett ML, Yapa C, Parag V, Anderson CS (2005). Frequency of depression after stroke: a systematic review of observational studies. Stroke.

[2] Campbell BC, Murray J, Holmes J, Astin F, Greenwood D, Knapp P (2013). Frequency of anxiety after stroke: a systematic review and meta-analysis of observational studies. Int J Stroke.

[3] Aström M (1996). Generalized anxiety disorder in stroke patients. A 3-year longitudinal study. Stroke.

[4] Aström M, Adolfsson R, Asplund K (1993). Major depression in stroke patients. A 3-year longitudinal study. Stroke.

[5] Frühwald S, Löffler H, Eher R, Saletu B, Baumhackl U (2001). Relationship between depression, anxiety and quality of life: a study of stroke patients compared to chronic low back pain and myocardial ischemia patients. Psychopathology.

[6] Intercollegiate Stroke Working Party (2012). National clinical guideline for stroke.

[7] Care Quality Commission (2011). Supporting life after stroke.

[8] Campbell BC, Holmes J, Murray J, Gillespie D, Lightbody C, Watkins C, Knapp P (2011). Interventions for treating anxiety after stroke. Cochrane Database Syst Rev.

[9] Hackett M, Anderson C, House A, Xia J (2008). Interventions for treating depression after stroke. Cochrane Database Syst Rev.

[10] Richards D, Richardson T (2012). Computer-based psychological treatments for depression: a systematic review and meta-analysis. Clin Psychol Rev.

[11] Reger MA, Gahm GA (2009). A meta-analysis of the effects of internet- and computer-based cognitive-behavioral treatments for anxiety. J Clin Psychol.

[12] Vallury KD, Jones M, Oosterbroek C (2015). Computerized cognitive behavior therapy for anxiety and depression in rural areas: a systematic review. J Med Internet Res.

[13] Kay-Lambkin FJ, Baker AL, Kelly B, Lewin TJ (2011). Clinician-assisted computerised versus therapist-delivered treatment for depressive and addictive disorders: a randomised controlled trial. Med J Aust.

[14] National Institute for Health and Clinical Excellence (2009). Depression in adults with a chronic physical health problem.

[15] Simblett SK, Ring H, Bateman A (2011). Computerised cognitive behavioural therapy (cCBT): a possible treatment for mood disorders experienced by people with neurological conditions?. Neuropsychol Rehabil.

[16] Gould RL, Coulson MC, Howard RJ (2012). Efficacy of cognitive behavioral therapy for anxiety disorders in older people: a meta-analysis and meta-regression of randomized controlled trials. J Am Geriatr Soc.

[17] Gould RL, Coulson MC, Howard RJ (2012). Cognitive behavioral therapy for depression in older people: a meta-analysis and meta-regression of randomized controlled trials. J Am Geriatr Soc.

[18] Crabb RM, Cavanagh K, Proudfoot J, Learmonth D, Rafie S, Weingardt KR (2012). Is computerized cognitive-behavioural therapy a treatment option for depression in late-life? a systematic review. Br J Clin Psychol.

[19] Barney LJ, Griffiths KM, Jorm AF, Christensen H (2006). Stigma about depression and its impact on help-seeking intentions. Aust N Z J Psychiatry.

[20] Layard R (2006). The case for psychological treatment centres. BMJ.

[21] Lancaster GA (2015). Pilot and feasibility studies come of age!. Pilot Feasibility Stud.

[22] Beck A, Steer R, Brown G (1996). Beck Depression Inventory-II manual.

[23] Beck A, Steer R (1993). Beck Anxiety Inventory manual.

[24] Proudfoot J, Ryden C, Everitt B, Shapiro DA, Goldberg D, Mann A, Tylee A, Marks I, Gray JA (2004). Clinical efficacy of computerised cognitive-behavioural therapy for anxiety and depression in primary care: randomised controlled trial. Br J Psychiatry.

[25] Simblett S, Craven J, Mercer J, Gracey F, Ring H, Bateman A (2011). Beating the blues after a stroke: a case presentation. http://www.babcpconference.com/archive/guildford2011/programme/abstracts_2011.pdf:.

[26] Benedict RH, Harris AE, Markow T, McCormick JA, Nuechterlein KH, Asarnow RF (1994). Effects of attention training on information processing in schizophrenia. Schizophr Bull.

[27] das Nair R, Moreton BJ, Lincoln NB (2011). Rasch analysis of the Nottingham extended activities of daily living scale. J Rehabil Med.

[28] Mioshi E, Dawson K, Mitchell J, Arnold R, Hodges JR (2006). The Addenbrooke's cognitive examination revised (ACE-R): a brief cognitive test battery for dementia screening. Int J Geriatr Psychiatry.

[29] Nelson H (1982). National adult reading test (NART) for the assessment of premorbid intelligence in patients with dementia: test manual.

[30] Topolovec-Vranic J, Cullen N, Michalak A, Ouchterlony D, Bhalerao S, Masanic C, Cusimano MD (2010). Evaluation of an online cognitive behavioural therapy program by patients with traumatic brain injury and depression. Brain Inj.

[31] Dobson KS, Hollon SD, Dimidjian S, Schmaling KB, Kohlenberg RJ, Gallop RJ, Rizvi SL, Gollan JK, Dunner DL, Jacobson NS (2008). Randomized trial of behavioral activation, cognitive therapy, and antidepressant medication in the prevention of relapse and recurrence in major depression. J Consult Clin Psychol.

[32] Hind D, O'Cathain A, Cooper CL, Parry GD, Isaac CL, Rose A, Martin L, Sharrack B (2010). The acceptability of computerised cognitive behavioural therapy for the treatment of depression in people with chronic physical disease: a qualitative study of people with multiple sclerosis. Psychol Health.

[33] Mohlman J, Gorman JM (2005). The role of executive functioning in CBT: a pilot study with anxious older adults. Behav Res Ther.

[34] Broomfield NM, Laidlaw K, Hickabottom E, Murray MF, Pendrey R, Whittick JE, Gillespie DC (2011). Post-stroke depression: the case for augmented, individually tailored cognitive behavioural therapy. Clin Psychol Psychother.

[35] Kaltenthaler E, Parry G, Beverley C, Ferriter M (2008). Computerised cognitive-behavioural therapy for depression: systematic review. Br J Psychiatry.

[36] Andrewes H, Kenicer D, McClay C, Williams C (2013). A national survey of the infrastructure and IT policies required to deliver computerised cognitive behavioural therapy in the English NHS. BMJ Open.

[37] Montero-Marín J, Prado-Abril J, Botella C, Mayoral-Cleries F, Baños R, Herrera-Mercadal P, Romero-Sanchiz P, Gili M, Castro A, Nogueira R, García-Campayo J (2015). Expectations among patients and health professionals regarding Web-based interventions for depression in primary care: a qualitative study. J Med Internet Res.

[38] Du E, Quayle E, Macleod H (2013). Service providers' perceptions on the uptake of computerised cognitive behavioural therapy (CCBT). PsychNology Journal.

[39] Sundram F, Hawken SJ, Stasiak K, Lucassen MF, Fleming T, Shepherd M, Greenwood A, Osborne R, Merry SN (2017). Tips and traps: lessons from Codesigning a clinician e-monitoring tool for computerized cognitive behavioral therapy. JMIR Ment Health.

