# Supplementary material for: Computerized Cognitive Behavioral Therapy to Treat Emotional Distress After Stroke: A Feasibility Randomized Controlled Trial
Source: JMIR Ment Health. 2017 May 31;4(2):e16. doi: 10.2196/mental.6022 (PMC5471343; doi:10.2196/mental.6022)
Supplement: Multimedia Appendix 1 [file mental_v4i2e16_app1.pdf]

Table 1. Summary of the structure and content included in the two interventions.

Module	cCBT: Beating the Blues		cCRT: ForemenRehab
1	Problem definition and identifying pleasurable events<br>Identifying thinking errors and distraction techniques		Introduction to attention and areas of the brain involved, followed by attention training exercises
2	*Cognitive*	*Behavioral*	Introduction to memory and areas of the brain involved, followed by memory training exercises
	Identifying negative automatic thoughts	Choice of<br>Scheduling pleasurable events<br>*or*<br>Structured problem solving	
3	Introduction to thinking errors and distraction techniques		Introduction to visual perception and areas of the brain involved, followed by visual perception training exercises
4	Evaluating negative automatic thoughts	Switch to learning remaining technique<br>Scheduling pleasurable events<br>*or*<br>Structured problem solving	Introduction to executive functioning and areas of the brain involved, followed by executive functioning training exercises
5			Choice of<br>Attention<br>Memory<br>Visual perception<br>*and/or*<br>Executive functioning<br>to suit individual need
6	Identifying and evaluating core beliefs	Choice of<br>Graded exposure to anxiety-provoking stimuli<br><br>Structured ways of breaking tasks down<br><br>*or*<br>Sleep management	
7	Attributional style		
8	Action planning and conclusions		
